# RvE1 Attenuates Polymicrobial Sepsis-Induced Cardiac Dysfunction and Enhances Bacterial Clearance

**DOI:** 10.3389/fimmu.2020.02080

**Published:** 2020-09-02

**Authors:** Jianmin Chen, Gareth S. D. Purvis, Debora Collotta, Sura Al Zoubi, Michelle A. Sugimoto, Antonino Cacace, Lukas Martin, Roman A. Colas, Massimo Collino, Jesmond Dalli, Christoph Thiemermann

**Affiliations:** ^1^Barts and the London School of Medicine and Dentistry, William Harvey Research Institute, Queen Mary University of London, London, United Kingdom; ^2^Sir William Dunn School of Pathology, University of Oxford, Oxford, United Kingdom; ^3^Department of Drug Science and Technology, University of Turin, Turin, Italy; ^4^Department of Basic Medical Sciences, School of Medicine, Al-Balqa Applied University, As-Salt, Jordan; ^5^Diabetes Complication Research Centre, School of Medicine, UCD Conway Institute, University College Dublin, Dublin, Ireland; ^6^Department of Intensive Care and Intermediate Care, RWTH University Hospital Aachen, Aachen, Germany

**Keywords:** polymicrobial sepsis, resolvin E1, bacterial clearance, immune response, cardiomyopathy

## Abstract

The development of cardiac dysfunction caused by microbial infection predicts high mortality in sepsis patients. Specialized pro-resolving mediators (SPMs) mediate resolution of inflammation in many inflammatory diseases, and are differentially expressed in plasma of sepsis patients. Here, we investigated whether the levels of SPMs are altered in the murine septic heart following polymicrobial sepsis-induced cardiac dysfunction. Ten weeks-old male C57BL/6 mice were subjected to polymicrobial sepsis induced by cecal ligation and puncture (CLP), which is a clinically relevant sepsis model receiving analgesics, antibiotics, and fluid resuscitation. CLP caused a significant systolic dysfunction assessed by echocardiography. The hearts were subjected to LC-MS/MS based lipid mediator profiling. Many SPMs were significantly reduced in septic hearts, among which RvE1 had a ~93-fold reduction. Treatment of CLP mice with synthetic RvE1 (1 μg/mouse *i.v*.) at 1 h after CLP increased peritoneal macrophages number, particularly MHC II^−^ macrophages. RvE1 reduced pro-inflammatory gene expression (interleukin-1β, interleukin-6, and CCL2) in lipopolysaccharide-stimulated bone marrow-derived macrophages (BMDMs) *in vitro*. RvE1 attenuated cardiac dysfunction in septic mice and increased cardiac phosphorylated Akt; decreased cardiac phosphorylated IκB kinase α/β, nuclear translocation of the NF-κB subunit p65, extracellular signal–regulated kinase 1/2, and c-Jun amino-terminal kinases 1/2. Most notably, RvE1 treatment reduced peritoneal bacterial load and promoted phagocytosis activity of BMDMs. In conclusion, cardiac SPMs, particularly RvE1, are substantially reduced in mice with polymicrobial sepsis. Delayed therapeutic administration of RvE1 to mice with polymicrobial sepsis attenuates the cardiac dysfunction through modulating immuno-inflammatory responses. In addition to the above effects, the ability to enhance bacterial clearance makes RvE1 an ideal therapeutic to reduce the sequalae of polymicrobial sepsis.

## Introduction

Sepsis is a lethal syndrome caused by a systemic dysregulation of host response to a bacterial, viral or fungal infection (1). About 50% of patients with sepsis develop cardiac dysfunction (2). The presence of cardiac dysfunction causes the mortality rate in septic patients to increase by 40–70% (3). The development of cardiac dysfunction in septic patients is associated with increased levels of pro-inflammatory cytokines, however, treatment targeting pro-inflammatory mediators in sepsis patients has had limited success (4). Moreover, the previously reported benefit of “early goal-directed therapy” providing cardiovascular support to patients with sepsis (5) has not been reproduced in multicenter clinical trials (6). Thus, novel pharmacological strategies which reduce sepsis-induced cardiac (organ) dysfunction, while enhancing bacterial clearance are urgently needed.

Resolution of inflammation is a physiological, active programme to terminate inflammation and enable homeostasis, coordinated by a number of endogenous specialized pro-resolving mediators (SPMs), including resolvins (Rvs), protectins, maresins (MaRs), and lipoxins (LXs) (7). Recently, we have reported that patients with sepsis showed differentially expressed SPMs; this was correlated with survival and acute respiratory distress syndrome development (8).

Rvs, including E-series (RvE) and D-series resolvins, are derived from ω-3 polyunsaturated fatty acids eicosapentaenoic acid (EPA) and docosahexaenoic acid, respectively (9). In particular, RvE1 is biosynthesized from EPA *via* either aspirin-acetylated cyclooxygenase-2 (COX-2) or cytochrome P450 monooxygenase in combination with 5-lipoxygenase (10). RvE1 potently promotes the resolution of inflammation in many animal models of inflammatory diseases, such as periodontal disease (11), allergic airway inflammation (12), and bacterial pneumonia (13). However, the role of RvE1 or other SPMs in the polymicrobial sepsis-induced cardiac dysfunction is unknown. Thus, the aim of the present study is to analyze the levels of SPMs in the heart tissue in mice with sepsis. Having found that septic challenge leads to substantial reductions of multiple SPMs, particularly RvE1 (~93-fold), we have then investigated the potential beneficial effects of the delayed therapeutic administration of synthetic RvE1 in a clinically relevant polymicrobial sepsis model in mice receiving analgesics, antibiotics, and fluid resuscitation.

## Materials and Methods

### Animals

The local “Animal Use and Care Committee” approved animal experiments in accordance with the derivatives of both, the “Home Office guidance on the Operation of Animals (Scientific Procedures) Act 1986,” and the “Guide for the Care and Use of Laboratory Animals” of the National Research Council. This study was carried out on 49 ten weeks-old male C57BL/6 mice (Charles River, Kent, UK), receiving a standard diet and water *ad-libitum*.

### Model of Polymicrobial Sepsis Caused by Cecal Ligation and Puncture (CLP) and RvE1 Treatment

CLP surgery was conducted in mice as described previously (14, 15). Based on previous evidence and preliminary data, an 18-G needle was used with the double puncture technique in order to generate reproducible cardiac dysfunction during the early phase of sepsis (24 h). Briefly, anesthesia was induced with 3% isoflurane and maintained at 2% for the duration of the surgery. Buprenorphine (0.05 mg/kg *i.p*.) was injected additionally to provide adequate analgesia. The rectal temperature of the animals was maintained at 37°C with a homeothermic blanket. The abdomen was opened *via* a 1.5 cm midline incision, and the cecum exposed. The cecum was ligated just below the ileocecal valve and punctured at both opposite ends. After a small amount of fecal matter was extruded from both ends, the cecum was placed back in its anatomical position and the abdomen was sutured. Ringer's solution was given *s.c*. for resuscitation directly after surgery (1 ml/mouse) and 6 h and 18 h after surgery (0.5 ml/mouse). Antibiotic (Imipenem/Cilastin; 20 mg/kg *s.c*.) and analgesia (buprenorphine; 0.05 mg/kg *i.p*.) was administered 6 and 18 h after surgery. Sham-operated mice were not subjected to ligation or perforation of cecum but were otherwise treated the same way. One hour after CLP or sham-operation, mice were treated either with RvE1 (1 μg/mouse *i.v*.; CAS number 552830-51-0; Cayman Chemical) (16) or vehicle (100 μl PBS, 0.1% Ethanol). A clinical score for monitoring the health of experimental mice was used to evaluate the symptoms consistent with murine sepsis. The maximum score of 6 comprised the presence of the following signs: lethargy, piloerection, tremors, periorbital exudates, respiratory distress, and diarrhea. Mice with a clinical score >3 were defined as exhibiting severe sepsis, against a moderate sepsis for a score ≤3. At 24 h after surgical procedures, cardiac function was assessed by echocardiography *in vivo*. Mice were then deeply anesthetized with 3% isoflurane, blood was taken by cardiac puncture, and mice were euthanized with CO_2_. Heart samples were stored at−80°C for further analysis.

To generate heart samples for lipid mediator qualification, mice were randomly allocated into two different groups: (i) Sham-operation (*n* = 5); (ii) CLP (*n* = 7).

In RvE1 therapeutic study for cardiac function analysis, mice were randomly allocated into four different groups: (i) Sham-operation + Vehicle (*n* = 6); (ii) Sham-operation + RvE1 (1 μg/mouse, *n* = 6); (iii) CLP + Vehicle (*n* = 8); (iv) CLP + RvE1 (1 μg/mouse, *n* = 8).

### Assessment of Cardiac Function *in vivo*

Cardiac function was assessed in mice by echocardiography *in vivo* as reported previously (14, 17). At 24 h after CLP, anesthesia was induced with 3% isoflurane and maintained at 0.5–0.7% for the duration of the procedure. Before assessment of cardiac function, mice were allowed to stabilize for at least 10 min. During echocardiography the heart rate was obtained from electrocardiogram (ECG) tracing and the temperature was monitored with a rectal thermometer. Two-dimensional and M-mode echocardiography images were recorded using a Vevo-770 imaging system (VisualSonics, Toronto, Ontario, Canada). Percentage fractional area change (FAC) was assessed from a two-dimensional trace and percentage ejection fraction (EF) and percentage fractional shortening (FS) were calculated from the M-mode measurements in the parasternal short axis view at the level of the papillary muscles. Calculation of percentage EF and FS requires the measurements of left ventricle internal dimension (LVID) in diastolic and systolic phase from M-mode, and calculation of FAC requires the measurements of LV end-systolic and end-diastolic areas from the B-mode.

### Lipid Mediator Qualification

After cardiac function analysis, mice were then deeply anesthetized with 3% isoflurane, and were euthanized with CO_2_. Hearts were placed in ice cold MeOH containing deuterated internal standards (d_4_-LTB_4_, d_8_-5S-HETE, d_4_-PGE_2_, d_5_-LXA_4_, and d_5_-RvD2, 500 pg each). These were gently homogenized, kept at −20°C for 45 min to allow for protein precipitation the subjected to solid phase extraction (18). Methyl formate fractions were brought to dryness using a TurboVap LP (Biotage) and products suspended in water-methanol (50:50 vol:vol) for LC-MS/MS. Here a Shimadzu LC-20AD HPLC and a Shimadzu SIL-20AC autoinjector (Shimadzu, Kyoto, Japan), paired with a QTrap 5500 (ABSciex, Warrington, UK) was utilized and operated as described previously (18). To monitor each LM and respective pathways, an MRM method was developed with diagnostic ion fragments (Table S1) and identification using recently published criteria (18), including matching retention time (RT) to synthetic and authentic materials and at least six diagnostic ions for each LM. Calibration curves were obtained for each using authentic compound mixtures and deuterium labeled LM at 3.12, 6.25, 12.5, 25, 50, 100, and 200 pg. Linear calibration curves were obtained for each LM, which gave *r*^2^ values of 0.98–0.99.

### Western Blot Analysis

Mice were euthanized with CO_2_ as described above. Semi-quantitative western blot analysis was carried out in mouse heart tissues as described previously (19, 20).

Briefly, mouse heart samples were homogenized in 10% homogenization buffer and centrifuged at 1,300 × g for 5 min at 4°C. Supernatants were removed and centrifuged at 16,000 × g at 4°C for 40 min to obtain supernatant containing the cytosolic fraction. The pelleted nuclei were re-suspended in extraction buffer (1/3 volume of the homogenation buffer) containing 20 mM HEPES (pH 7.9), 1.5 mM MgCl2, 420 mM NaCl, 0.2 mM EDTA, 20% glycerol, 1 mM EGTA (and inhibitor of protease) and incubated in ice for 30 min, followed by centrifugation at 16,000 g for 20 min at 4°C. The resulting supernatants containing nuclear proteins were carefully removed, and protein content was determined on both nuclear and cytosolic extracts using a bicinchoninic acid (BCA) protein assay following the manufacturer's directions (Therma Fisher Scientific, Rockford, IL). Proteins were separated by 8% sodium dodecyl sulphatepolyacrylamide gel electrophoresis (SDS-PAGE) and transferred to a polyvinyldenedifluoride (PVDF) membrane, which were blocked with a solution of 5% dry milk in TBS-Tween for 2 h. Membranes were the incubated, overnight, with a primary antibody: rabbit anti-total inhibitor of kappa B kinase (IKK) α/β; rabbit anti-phospho-IKKα/β Ser^176/180^; rabbit anti-NF-κB p65; rabbit anti-total Akt; mouse anti-phospho-Akt pSer^473^; rabbit anti-total p44/42 MAPK (Erk1/2); rabbit anti-phospho-p44/42 MAPK (Erk1/2) (Thr202/Tyr204); rabbit anti-total-SAPK/JNK, mouse anti-phospho-SAPK/JNK (Thr183/Tyr185) were used at 1:1000 dilution. Blots were then incubated with a secondary antibody conjugated with horseradish peroxidase (dilution 1:10000) for 30 min at room temperature and developed with the ECL detection system. The immunoreactive bands were visualized by autoradiography. Densitometric analysis of the bands was performed using the Bio-Rad Image Lab TM 6.0.1 Software. Each group was then adjusted against corresponding sham data to establish relative protein expression when compared with sham animals.

### Bacteria Counting

The mice were euthanized with CO_2_ at 24 h after the CLP. The skin of the abdomen was cut open after disinfection and without damage to the muscle layer, the peritoneal cavity was washed with 4 mL of sterile PBS with 2 mM EDTA. Aliquots of serial log dilutions of the obtained peritoneal lavage were plated on LB Broth with agar plates, colony-forming units (CFU) were counted after incubation in humidified incubator at 37°C for 24 h, the results were expressed as the number of CFU per ml.

### Flow Cytometry

Mice were euthanized with CO_2_ as described above. Peritoneal exudate was taken from four CLP + Vehicle and five CLP + RvE1 mice. Peritoneal cells were differentiated using anti-CD11b (clone M1/70; eBioscience), anti-F4/80 (clone BM8; BioLegend), anti-Ly6G (clone 1A8; BioLegend), anti-I-A/I-E (clone M5/114.15.2; BioLegend), anti-Ly6C (clone HK1.4; BioLegend), anti-CD64 (clone X54-5/7.1; BioLegend) antibodies. Zombie NIR™ Fixable Viability Kit (Biolegend) was used to identify live cells. Cell counts were determined using Precision Count Beads (Biolegend). Ten thousand live cell events were acquired with a FACSCalibur (Becton Dickinson) and analyzed using FlowJo analysis software (version 9.2, Treestar Inc.).

### Bone Marrow-Derived Macrophages (BMDMs)

Bone marrow derived macrophages were generated as previously described (21). Briefly, Male C57BL/6J mice were euthanized with CO_2_. Tibiae and femurs from these mice were flushed with PBS and bone marrow cells were re-suspended in Dulbecco Modified Eagle's Medium supplemented with 10% heat-inactivated FBS, 10% L929-conditioned medium (which is a source of M-CSF) and 1% Penicillin-Streptomycin. Cells were cultured for seven days at 37°C/5% CO_2_ and on day 3 media was changed (only adherent cells were left in culture).

### IncuCyte Zoom Phagocytosis

BMDMs treated with either vehicle or 10 nM RvE1 for 30 min were then incubated with unopsonized pHrodo Green *E. coli* Bioparticles (ThermoFisher Scientific, Loughborough, UK) at 0.1 mg/ml in a 96-well flat clear bottom plate. The plate was then placed into the IncuCyte Zoom platform (Essen Bioscience, Welwyn Garden City, UK) which was housed inside a humidified incubator at 37°C, 5% CO_2_. Two to four images per well from three technical replicates were taken every 20 min for 5 h using a 20× objective lens and then analyzed using the IncuCyte™ Basic Software. Green channel acquisition time was 400 ms.

### mRNA Expression Analysis

BMDMs were pre-treated with 10 nM RvE1 for 30 min followed by 2 h lipopolysaccharide (LPS) (0.1 μg/ml) stimulation. BMDMs mRNA was extracted with TRIzol reagent (Thermo Fisher Scientific), and total RNA concentration and quality were determined with a ND-1000 spectrophotometer (Nano Drop Technologies, Wilmington, DE, USA). cDNA was synthesized from 1,000 ng RNA using the QuantiTect Reverse Transcription kit (Qiagen, Manchester, UK) according to the manufacturer's instructions. Real-time quantitative PCR was performed using either Taqman or Sybr Select gene expression master mix (Life Technologies) in the StepOnePlus™ thermal cycler (Applied Biosystems). Primers were purchased from Qiagen (tnfα, il-6, ccl2, il-1b, and 18S). Cycle threshold values were determined by the StepOne software and target gene expression was normalized to housekeeping gene (βactin). Relative expression results were plotted as mRNA expression divided by 18S expression.

### Statistics

All values described in the text and figures are presented as mean ± standard error of the mean (SEM) of *n* observations, where *n* represents the number of animals studied. Statistical analysis was performed using GraphPad Prism 7.0 (GraphPad Software, San Diego, California, USA). One-way ANOVA followed by Bonferroni's *post-hoc* test or unpaired Student's *t*-test were used to compare intergroup differences. Comparing results were considered statistically significant when *P* < 0.05. Partial Least Square Discriminant Analysis were performed using MetaboAnalyst (22). Here, features with a constant or single value across samples were deleted. Partial Least Square Discriminant Analysis was then performed following auto-scaling (mean-centered and divided by the standard deviation of each variable). PLS-DA is based on a linear multivariate model that identifies variables that contribute to class separation of observations on the basis of their variables (LM levels). During classification, observations were projected onto their respective class model. The score plot illustrates the systematic clusters among the observations (closer plots presenting higher similarity in the data matrix).

### Materials

All materials, reagents, and solutions were purchased from Sigma Aldrich (Poole, Dorset, UK), unless otherwise stated. Antibodies for immunoblot analysis were purchased from Cell Signaling Antibodies.

## Results

### SPMs, Particularly RvE1, Levels Were Substantially Reduced in Hearts From Mice With Polymicrobial Sepsis

**Figure 1:**
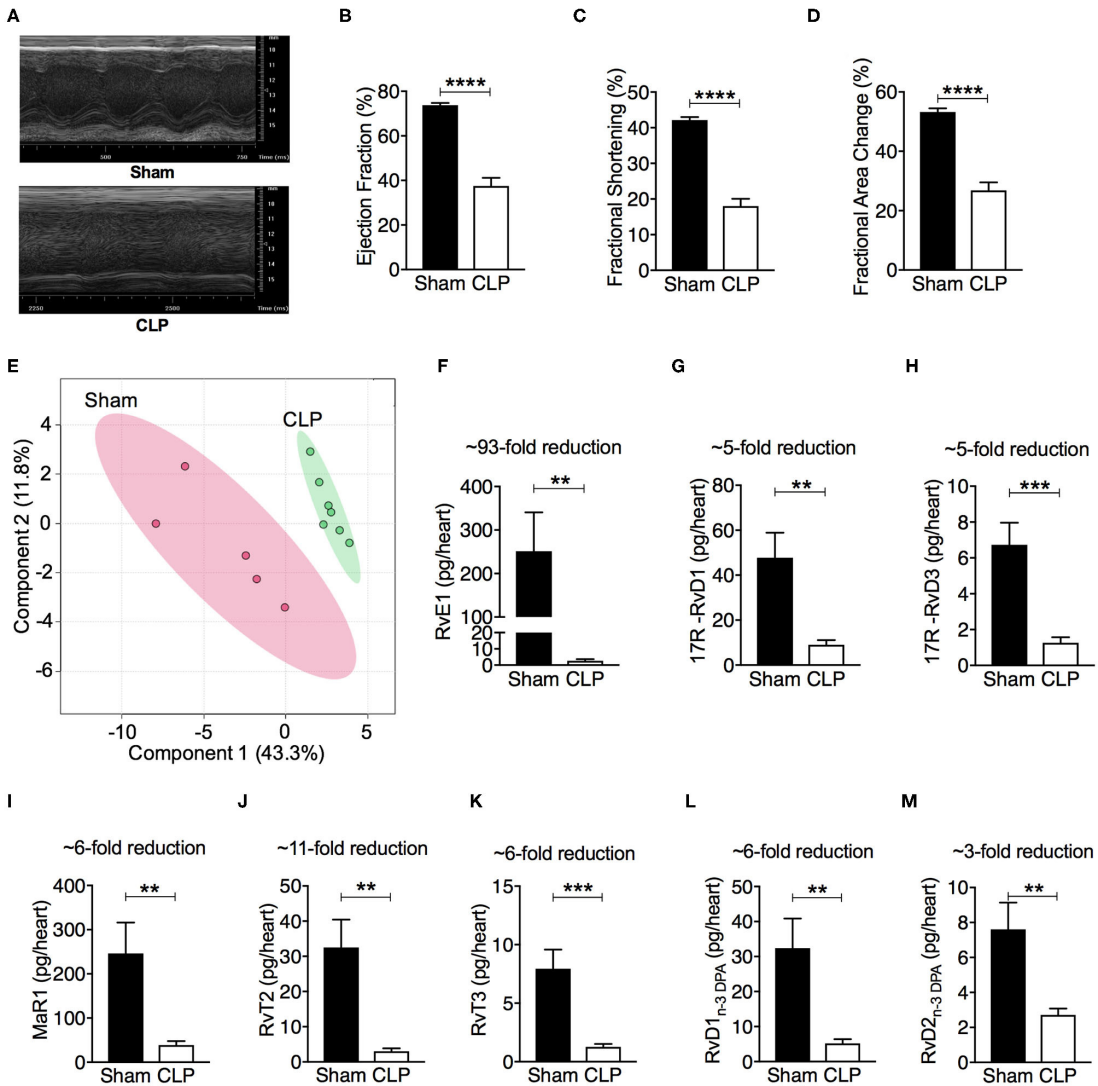
CLP causes significant cardiac dysfunction, and leads to a shift in heart tissue lipid mediator profiles and a downregulation of SPMs. **(A–D)** Cardiac function was assessed at 24 h after sham or CLP surgery. **(A)** Representative M–mode echocardiograms and percentages of **(B)** ejection fraction **(C)** fractional shortening, and **(D)** fractional area change. **(E–M)** Mice were subjected to CLP or sham surgery and heart tissues were collected after 24 h. Tissues were then homogenized and mediators were identified and quantified using LC-MS/MS based lipid mediator profiling. **(E)** Lipid mediator concentrations between the two groups were then interrogated using Partial Least Square Discriminant Analysis. Score Plot, with each dot representing profiles from each mouse. Cumulative levels for select SPMs including **(F)** RvE1, **(G)** 17R -RvD1, **(H)** 17R -RvD3, **(I)** MaR1, **(J)** RvT2, **(K)** RvT3, **(L)** RvD1_n−3DPA_, and **(M)** RvD2_n−3DPA_ in mouse hearts subjected to sham or CLP surgery. Results are expressed in pg/heart. **(B,C,F–M)** Data are represented as means ± SEM and were analyzed by unpaired Student's *t*-test. **P < 0.01, ***P < 0.001, ****P < 0.0001 vs. Sham-operated group. The following groups were studied: Sham-operated (*n* = 5) and CLP (*n* = 7). Rv, resolvin; MaR, maresin; LX, lipoxin.

CLP-induced polymicrobial sepsis caused significant cardiac dysfunction indicated by reductions in percentage EF, FS and FAC (*P* < 0.05; Figures 1A–D). To investigate whether this polymicrobial sepsis-induced cardiac dysfunction was associated with changes in pro-resolving mediators, hearts were harvested from mice following CLP or sham surgery. The profiles of pro-resolving mediators for the eicosapentaenoic acid and docosahexaenoic acid bioactive metabolomes in the mouse heart were determined by LC-MS/MS based lipid mediator profiling. We identified mediators from all four pro-resolving mediator families including resolvins, protectins, and maresins (Table S1), in accordance with published criteria (18). Principle component analysis of total lipid mediators demonstrated a marked shift in lipid mediator profiles in cardiac tissue from CLP mice when compared with tissues obtained from sham mice (Figure 1E). This shift was linked with a significant reduction in levels of multiple pro-resolving mediators including RvE1, 17R -RvD1, 17R -RvD3, MaR1, RvT2, RvT3, RvD1_n−3DPA_, RvD2_n−3DPA_, and LXB_4_, etc (Figures 1F–M, Table S1), among which the levels of RvE1 were the most reduced (sham: 251.9 ± 104.3 vs. CLP: 2.7 ± 1.0 pg/heart; ~93-fold reduction; *P* < 0.05). These findings led us to postulate that reductions in the cardiac levels of SPMs, particularly RvE1, may play a pivotal role in the pathophysiology of the cardiac dysfunction in mice with polymicrobial sepsis.

### RvE1 Treatment Attenuated Cardiac Dysfunction and Improved Clinical Outcome in Mice With Polymicrobial Sepsis

**Figure 2:**
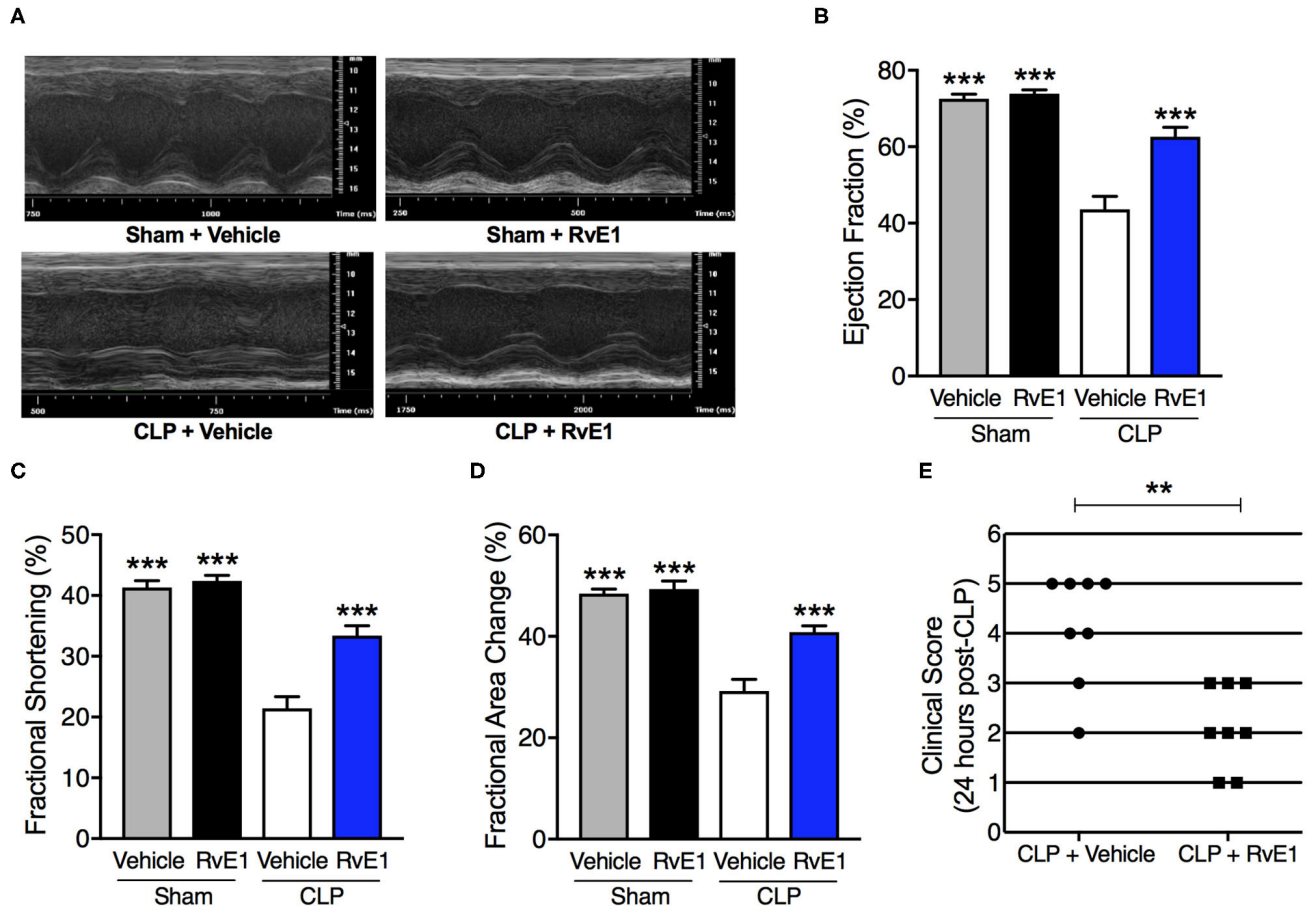
One hour post-treatment of RvE1 attenuates CLP-induced cardiac dysfunction and sepsis-associated clinical scores in mice. Mice underwent sham or CLP surgery. One hour after CLP, mice were treated with either RvE1 (1 μg/mouse *i.v*.) or vehicle (100 μl PBS, 0.1% Ethanol). Cardiac function was assessed at 24 h. **(A)** Representative M–mode echocardiograms and percentages of **(B)** ejection fraction **(C)** fractional shortening, and **(D)** fractional area change. The following groups were studied: Sham + Vehicle (*n* = 6); Sham + RvE1 (*n* = 6); CLP + Vehicle (*n* = 8) and CLP + RvE1 (*n* = 8). All data are represented as means ± SEM. Data were analyzed by one-way ANOVA followed by Bonferroni *post-hoc* test. ***P < 0.001 vs. CLP + Vehicle group. **(E)** At 24 h post-CLP, mice were scored for the presence or absence of six different macroscopic signs of sepsis, namely, lethargy, piloerection, tremors, periorbital exudates, respiratory distress, and diarrhea. A clinical score >3 is considered as severe sepsis. Data are from 16 mice and were analyzed by unpaired Student's *t*-test. **P < 0.01 vs. CLP + Vehicle group.

Having found that RvE1 was substantially reduced in the heart following polymicrobial sepsis challenge, we investigated if treatment with synthetic RvE1 attenuated polymicrobial sepsis-induced cardiac dysfunction. When compared with sham surgery plus vehicle mice, sham surgery mice treated with RvE1 showed no significant alterations in percentage EF, FS, and FAC (*P* > 0.05; Figures 2A–D). When compared with sham surgery mice, mice underwent CLP surgery plus vehicle developed significant cardiac dysfunction indicated by significant reductions in percentage EF, FS, and FAC (*P* < 0.05; Figures 2A–D); they were significantly attenuated by delayed administration of RvE1 1 h after CLP (*P* < 0.05; Figures 2A–D). Additionally, when compared with CLP mice with vehicle administration, RvE1 treatment significantly improved clinical scores in CLP mice: At 24 h post-CLP, 75% CLP mice with vehicle administration recorded a score >3, indicating severe sepsis, whereas 100% of RvE1-treated CLP mice developed moderate sepsis (score, ≤3) (Figure 2E).

### RvE1 Treatment Improved the Host Immune Response to Polymicrobial Sepsis

**Figure 3:**
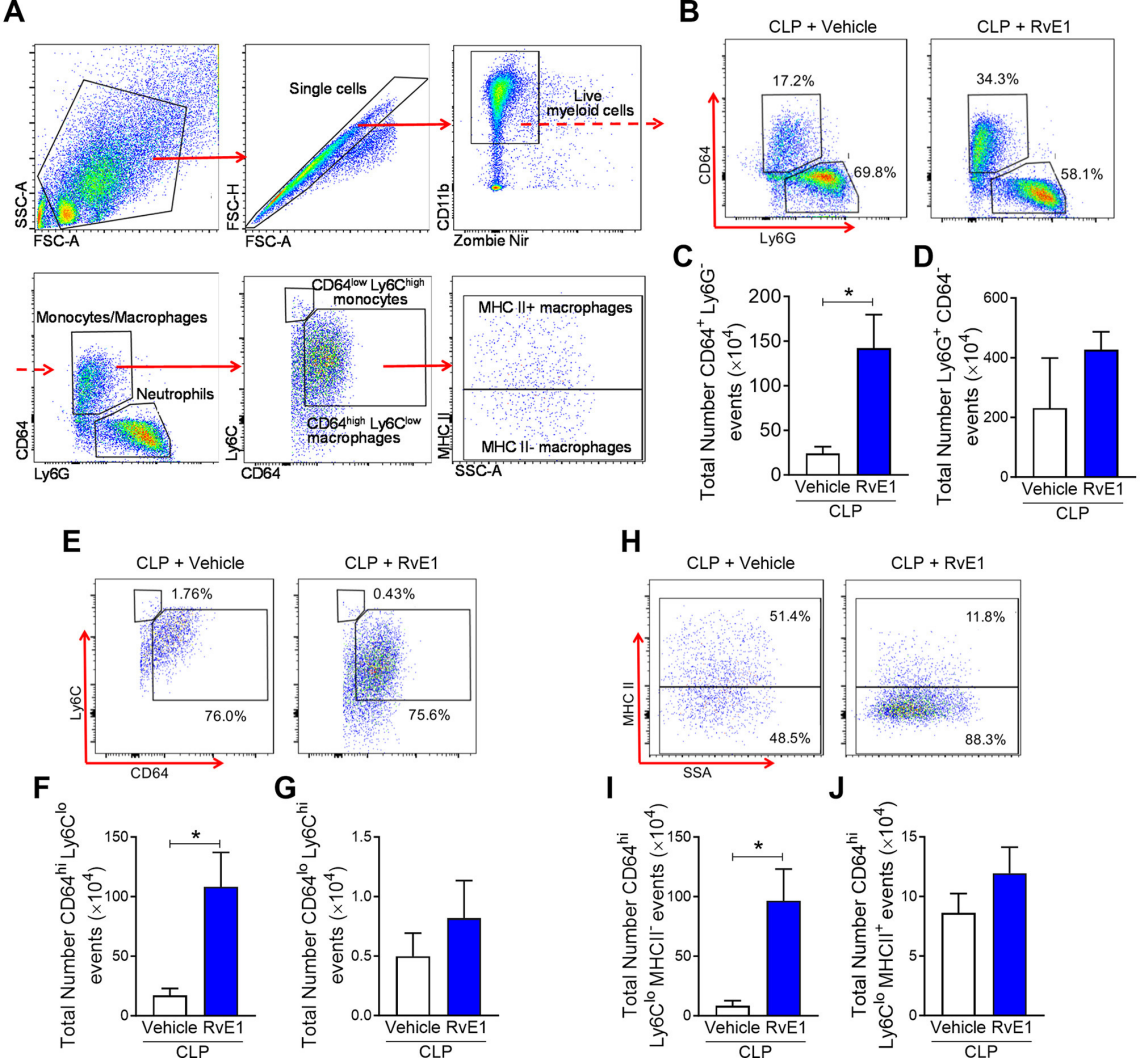
RvE1 treatment enhances MHC II^−^ macrophage recruitment in peritoneal cavity. Mice underwent CLP surgery. One hour after CLP, mice were treated with either RvE1 (1 μg/mouse *i.v*.) or vehicle (100 μl PBS, 0.1% Ethanol). **(A)** Flow cytometry gating strategy of mouse peritoneal immune cells 24 h post-CLP. **(B)** Scattergrams illustrating monocyte/macrophage (identified as Ly6G^−^CD64^+^) and neutrophil (identified as Ly6G^+^CD64^−^) positive events in peritoneal lavages from CLP mice with vehicle or RvE1 treatment. **(C,D)** Cumulative data for peritoneal CD64^+^ monocytes/macrophages and Ly6G^+^ neutrophils. **(E)** Scattergrams illustrating macrophage (identified as CD64^high^Ly6C^low^) and monocyte (identified as CD64^low^Ly6C^high^) positive events in peritoneal lavages from CLP mice with vehicle or RvE1 treatment. **(F,G)** Cumulative data for peritoneal CD64^high^Ly6C^low^ macrophages and CD64^low^Ly6C^high^ monocytes. **(H)** Scattergrams illustrating MHC II^−^ macrophage and MHC II^+^ macrophage positive events in peritoneal lavages from CLP mice with vehicle or RvE1 treatment. **(I,J)** Cumulative data for peritoneal MHC II^−^ macrophages and MHC II^+^ macrophages. Data are expressed as mean ± SEM of four mice for vehicle group and five mice for RvE1 treatment group. Data were analyzed by unpaired Student's *t*-test. *P < 0.05 vs. CLP + Vehicle group.

Analysis of peritoneal exudates by flow cytometry (Figure 3A) showed a trend for increased (although not significant) peritoneal myeloid cells at 24 h post-CLP in RvE1-treated CLP mice compared to CLP mice with vehicle administration (CLP + vehicle: 2.79 ± 1.80 (×10^6^) vs. CLP + RvE1: 6.16 ± 0.66 (×10^6^); *P* = 0.09), this increase was mainly due to increased Ly6G^−^CD64^+^ monocytes/macrophages recruitment (*P* < 0.05; Figures 3B,C). No significant difference was observed in Ly6G^+^CD64^−^ neutrophils (*P* > 0.05; Figures 3B,D). Ly6G^−^CD64^+^ monocytes/macrophages were subclassified as CD64^low^Ly6C^high^ monocytes and CD64^high^Ly6C^low^ macrophages (23), the later subset was further subclassified as major histocompatibility complex (MHC) II^+^ and MHC II^−^ macrophages (23). When compared to mice that had CLP surgery plus vehicle, mice which underwent CLP surgery and were treated with RvE1 had significantly increased CD64^high^Ly6C^low^ macrophages within the peritoneum (*P* < 0.05; Figures 3E,F), this was mainly attributed to the increase in MHC II^−^ macrophages (*P* < 0.05; Figures 3H,I), while no significant change was observed in MHC II^+^ macrophages (*P* > 0.05; Figures 3H,J) or CD64^low^Ly6C^high^ monocytes (*P* > 0.05; Figures 3E,G).

### RvE1 Reduced mRNA Expression of Pro-inflammatory Proteins in BMDMs With LPS Stimulation *in vitro*

**Figure 4:**
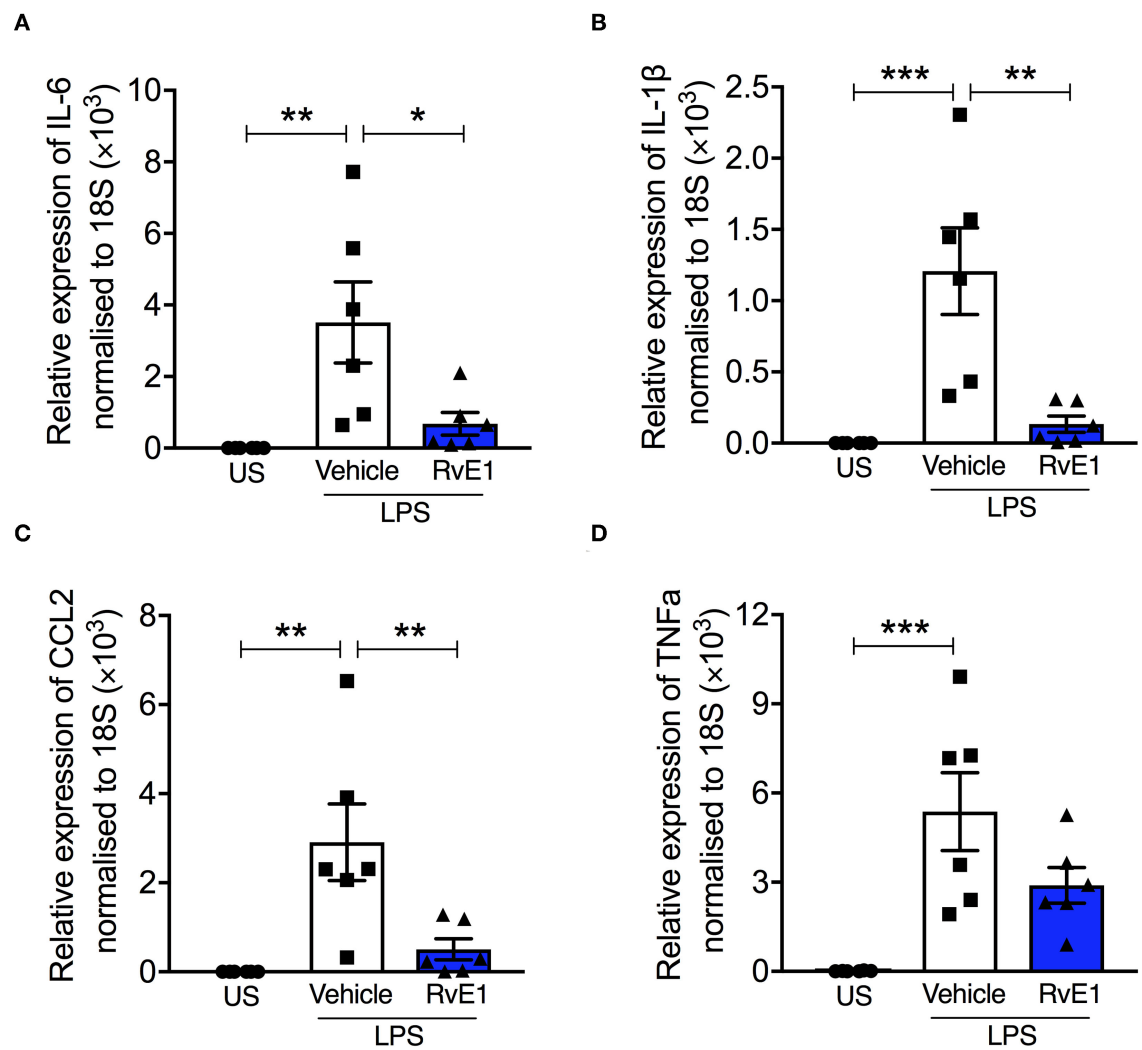
RvE1 reduces pro-inflammatory mRNA expression in BMDMs with LPS stimulation *in vitro*. BMDMs were treated with 10 nM RvE1 or vehicle after 2 h LPS (0.1 μg/ml) stimulation. Real-time quantitative PCR of **(A)** IL-6, **(B)** IL-1β, **(C)** CCL2, and **(D)** TNFα expression, relative to 18S rRNA. Each analysis **(A–D)** is from a single experiment and is representative of six separate experiments. Data are expressed as mean ± SEM. *P < 0.05, **P < 0.01, ***P < 0.001 vs. LPS + Vehicle group. US, unstimulated.

BMDMs stimulated with LPS (0.1 μg/ml) for 2 h resulted in significant increases in mRNA expression of pro-inflammatory cytokines and chemokines including IL-6, IL-1β, TNFα, and CCL2 (*P* < 0.05; Figures 4A–D). Pre-treatment with 10 nM RvE1 prior to LPS stimulation significantly attenuated the increases in IL-6, IL-1β, and CCL2 mRNA expression (*P* < 0.05; Figures 4A–C). There was a trend that TNFα mRNA expression was also reduced with RvE1 treatment compared with vehicle control (*P* = 0.09; Figure 4D).

### RvE1 Treatment Attenuated Changes in Cardiac Akt Phosphorylation, Activation of Nf-κB, and ERK1/2 and JNK1/2 Phosphorylation in Mice With Polymicrobial Sepsis

**Figure 5:**
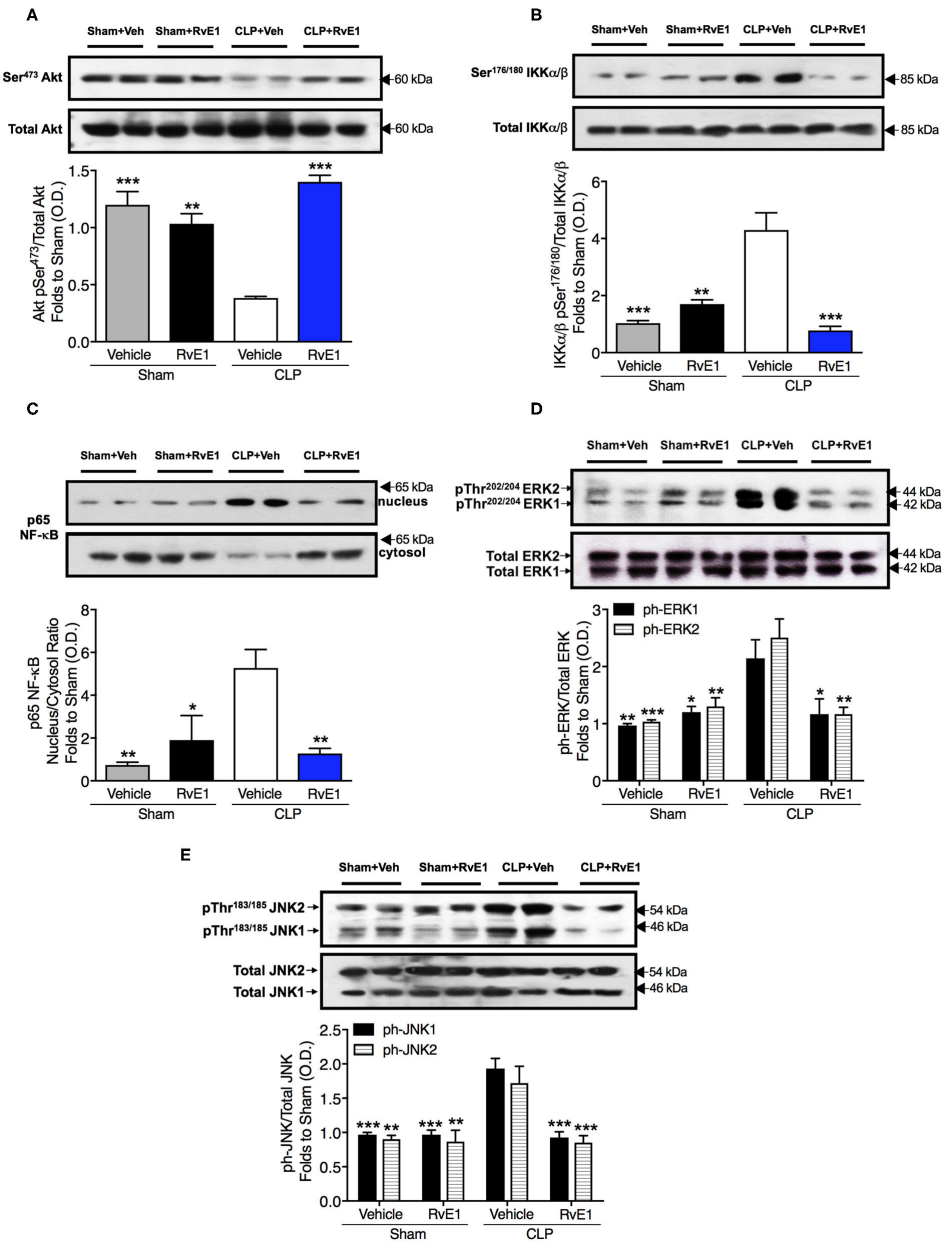
RvE1 improves cardiac phosphorylation of Akt and attenuates the increases in cardiac phosphorylation of IKKα/β, nuclear translocation of p65, ERK1/2, and JNK1/2 in CLP mice. Mice underwent sham or CLP surgery. One hour after CLP, mice were treated with either RvE1 (1 μg/mouse *i.v*.) or vehicle (100 μl PBS, 0.1% Ethanol). Signaling events in heart tissue were assessed at 24 h. Densitometric analysis of the bands is expressed as relative optical density (O.D.) of **(A)** phosphorylated Akt (pSer^473^) corrected for the corresponding total Akt content and normalized using the related sham band; **(B)** phosphorylated IKKα/β (pSer^176/180^) corrected for the corresponding total IKKα/β content and normalized using the related sham band; **(C)** NF-κB p65 subunit levels in both, cytosolic and nuclear fractions expressed as a nucleus/cytosol ratio normalized using the related sham bands; **(D)** ERK1/2 phosphorylation, corrected for the corresponding total ERK1/2 content and normalized using the related sham band; **(E)** JNK1/2 phosphorylation, corrected for the corresponding total JNK1/2 content and normalized using the related sham band. Each analysis **(A–E)** is from a single experiment and is representative of three to four separate experiments. Data are expressed as mean ± SEM for *n* number of observations. Data were analyzed by one-way ANOVA followed by Bonferroni's *post-hoc* test. *P < 0.05, **P < 0.01, ***P < 0.001 vs. CLP + Vehicle group.

To gain a better mechanistic insight into the cardioprotective effects of RvE1 in mice with polymicrobial sepsis, we investigated the effects of RvE1 treatment on the cardiac phosphorylation of Akt on Ser^473^, phosphorylation of IKKα/β on Ser^176/180^, subsequent nuclear translocation of p65 NF-κB, and phosphorylation of ERK1/2 on Tyr^202^ and Tyr^204^ and JNK1/2 on Thr^183^ and Thr^185^. RvE1 administration did not alter any of the above parameters in sham surgery mice (Figures 5A–E). When compared to sham surgery mice, CLP surgery mice given vehicle exhibited a significantly lower degree of cardiac phosphorylation of Akt on Ser^473^. In contrast, RvE1 treatment to CLP surgery mice significantly attenuated the decrease in cardiac phosphorylation of Akt on Ser^473^ (*P* < 0.05; Figure 5A).

When compared to sham surgery mice, CLP surgery mice given vehicle exhibited significantly increased cardiac phosphorylation of IKKα/β on Ser^176/180^, nuclear translocation of p65 NF-κB phosphorylation of ERK1/2 on Tyr^202^ and Tyr^204^ and JNK1/2 on Thr^183^ and Thr^185^. In contrast RvE1 treatment to CLP surgery mice significantly attenuated the increases in IKKα/β phosphorylation, nuclear translocation of p65, and phosphorylation of ERK1/2 and JNK1/2 (*P* < 0.05; Figures 5B–E).

### RvE1 Treatment Improved Bacterial Clearance *in vivo* and *in vitro*

**Figure 6:**
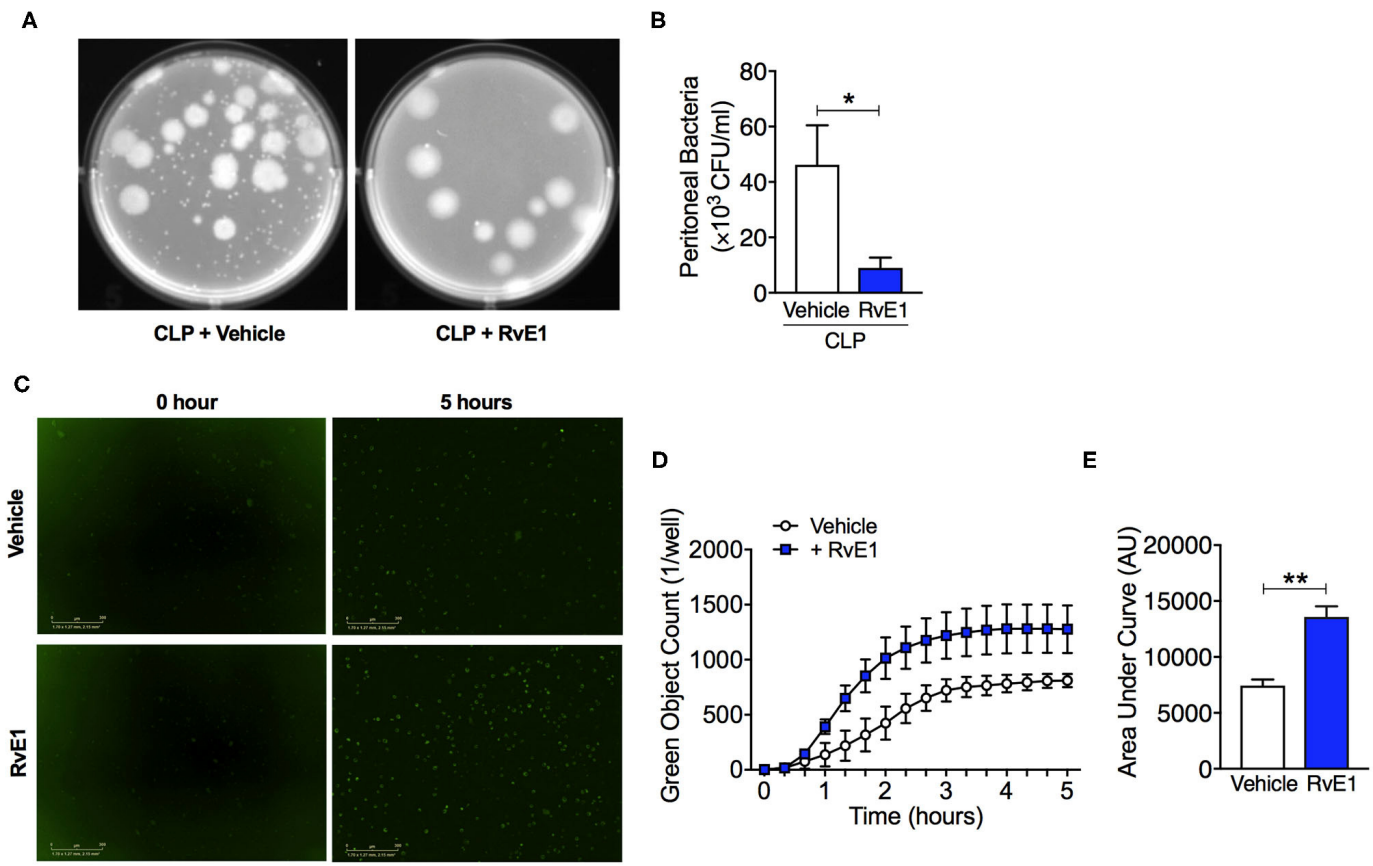
RvE1 treatment improves peritoneal bacterial clearance in mice with CLP, and promotes phagocytosis activity in BMDMs *in vitro*. **(A,B)** Mice underwent CLP surgery. One hour after CLP, mice were treated with either RvE1 (1 μg/mouse *i.v*.) or vehicle (100 μl PBS, 0.1% Ethanol). **(A)** Representative images of colony-forming units (CFU) and **(B)** Measurement of bacteria levels in peritoneal lavages 24 h post-CLP from mice with vehicle or RvE1 treatment. Data are expressed as mean ± SEM of six mice for vehicle group and five mice for RvE1 treatment group. Data were analyzed by unpaired Student's *t*-test. *P < 0.05 vs. CLP + Vehicle group. **(C–E)** BMDMs were pre-treated with RvE1 (10 nM) for 30 min, and then incubated with pH-rodo Green *E. coli* bioparticles (0.1 mg/ml), and fluorescence emission was measured in the IncuCyte imaging platform every 20 min for 5 h at 37°C. **(C)** Representative images from the IncuCyte software from one of *n* = 4 separate experiments are shown. Scale bar 200 μm. **(D)** The kinetics of phagocytosis at 20 min intervals. **(E)** Quantification of data in **(D)** showing area under the curve for 5 h following pH-rodo Green *E. coli* bioparticles addition. Data are presented as mean ± SEM from four biological replicates in technical duplicates. Data were analyzed by unpaired Student's *t*-test. **P < 0.01 vs. Vehicle group.

Uncontrolled bacterial load contributes to a worse outcome in sepsis (14), therefore improving bacterial clearance is of utmost importance for the treatment of sepsis. We therefore investigated whether RvE1 treatment could alter the bacterial clearance in mice with polymicrobial sepsis. When compared to CLP surgery mice given vehicle, RvE1-treated mice exhibited lower peritoneal bacterial load following CLP surgery as quantified by colony counts (*P* < 0.05; Figures 6A,B). As macrophages were one of the most abundant cell types present in the inflammatory exudates of mice post-CLP (Figure 3), we further investigated whether the phagocytic ability of BMDMs was improved following RvE1 treatment. We performed a pHrodo-conjugated *E. coli* phagocytosis assay using the IncuCyte ZOOM real-time cell imaging system as described previously (24). By taking images of the green fluorescence emitted by bacteria once inside the phagolysosome. BMDMs were pre-treated with 10 nM RvE1 30 min prior to the addition of *E. coli* bioparticles. RvE1 treatment greatly enhanced phagocytosis in BMDMs compared with vehicle treatment as reflected by the kinetics of phagocytosis (Figures 6C,D). Plotting the area under the curve of the kinetics traces (up to 5 h) illustrated that BMDMs treated with RvE1 had significantly increased green object counts compared with the control BMDM treated with vehicle (*P* < 0.05, Figure 6E). These data suggest that RvE1 enhances BMDMs phagocytosis of E. coli *in vitro*. Enhanced phagocytic ability of macrophages might contribute to the improved bacterial clearance in the peritoneal cavity of mice with polymicrobial sepsis treated with RvE1.

## Discussion

**Figure 7:**
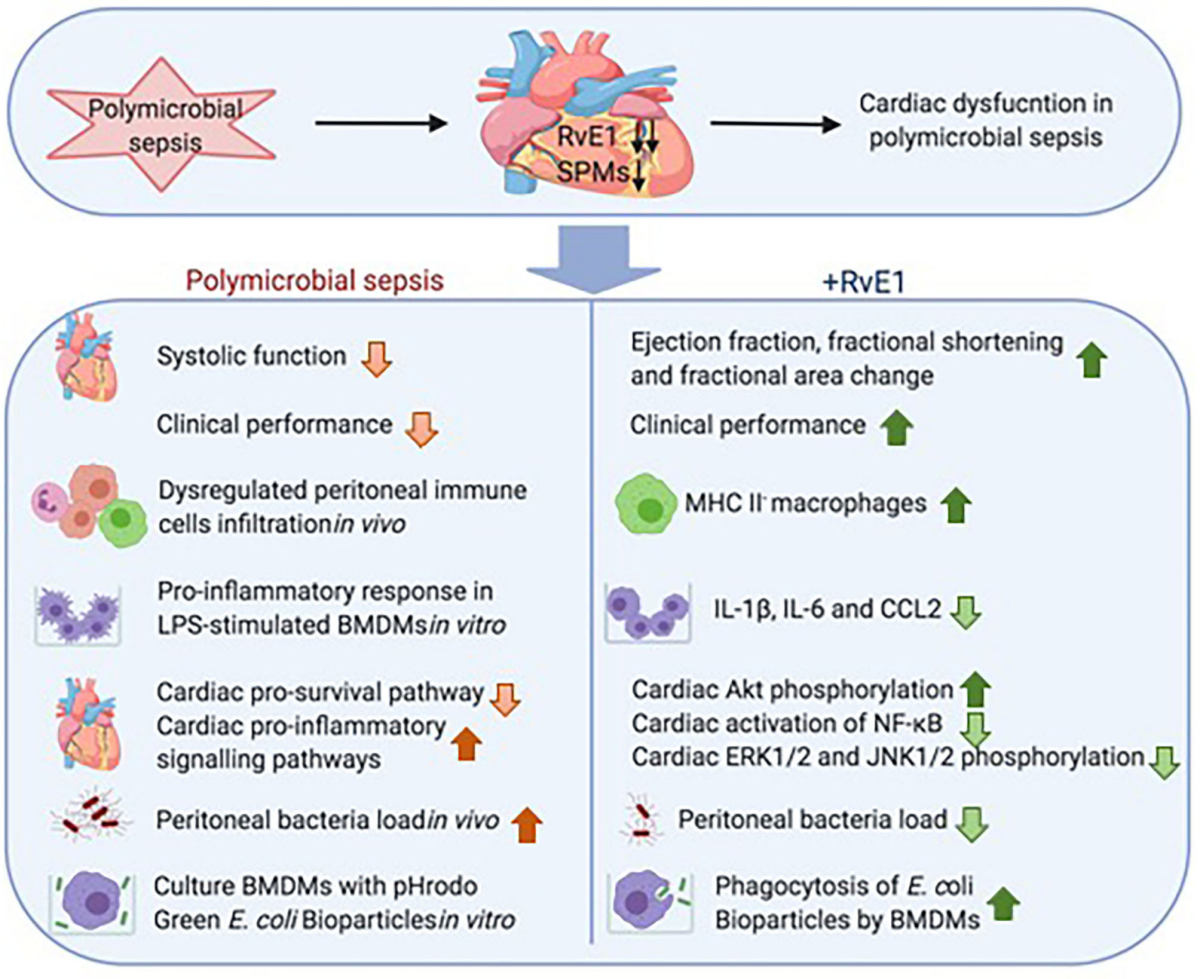
Schematic summary: RvE1 attenuates polymicrobial sepsis-induced cardiac dysfunction and enhances bacterial clearance. The upper panel displays substantial reduction of multiple SPMs, particularly RvE1, in the heart of mice with polymicrobial sepsis, and this reduction was associated with the development of profound cardiac dysfunction. The lower panel shows delayed therapeutic administration of RvE1 attenuated polymicrobial sepsis-induced cardiac dysfunction and augmented bacterial clearance, and its associated mechanisms as demonstrated *in vivo* and *in vitro*.

Sepsis is characterized by life-threatening organ dysfunction caused by a dysregulated host response to microbial infection. The presence of cardiac dysfunction in patients with sepsis has been linked to high morbidity and mortality rates, which together with the associated high socioeconomic costs (1) highlights an unmet need for identifying novel therapeutic strategies that attenuate sepsis-induced cardiac dysfunction. We report here for the first time that the cardiac dysfunction observed in septic mice is associated with significant reductions in several SPMs in the heart of septic mice. Particularly the levels of the pro-resolution mediator RvE1 were reduced by ~93-fold in the heart. As RvE1 is a potent pro-resolving mediator, which has clinical efficacy in humans with dry eye-associated inflammation (25) and has therapeutic effects in rodent models of myocardial infarction (26, 27) and LPS-induced cardiac injury (28), we hypothesized that reductions in endogenous levels of SPMs, particularly RvE1, may contribute to development of cardiac dysfunction in sepsis. To investigate this hypothesis, we have added back synthetic RvE1 to mice with polymicrobial sepsis-induced cardiac dysfunction. We report here for the first time that delayed treatment of RvE1 using a therapeutic approach (i.e., 1 h after CLP surgery) significantly attenuates the cardiac dysfunction and promotes bacterial clearance in polymicrobial sepsis (Figure 7).

We also observed an increased macrophages number, particularly MHC II^−^ macrophage subset in the peritoneum of septic mice treated with RvE1. MHC II^−^ macrophages express more M2-associated genes such as *CD36, CD163, Mertk*, and *Arg1*. While MHC II^+^ macrophages express more M1-associated genes such as *CD80, CD86*, and *Il1b* (23). Classically activated M1-like macrophages express pro-inflammatory cytokines (TNFa, IL-1β, and IL-6) and is involved in the initiation and maintenance of inflammation, whereas the alternatively activated M2-like macrophages express anti-inflammatory cytokines (IL-10, TGF-β) which drive resolution of inflammation, wound healing, and tissue repair (29). Interestingly, while we see an increase in macrophage number in mice treated with RvE1 following CLP surgery they do not appear to be MHC II^+^ macrophages, which have a pro-inflammatory phenotype, suggesting RvE1 treatment is reducing macrophage pro-inflammatory activation. Importantly, M2 macrophages produce higher levels of SPMs including RvE2, RvD5, MaR1, and LXA_4_ compared with M1, but lower levels of pro-inflammatory prostanoids and leukotrienes (30). Early-stage sepsis is associated with a hyperinflammatory response (31). Here we have shown that RvE1 increases macrophages number in the peritoneum, particularly MHC II^−^ macrophages suggesting that RvE1 treatment may induce a phenotypic switch in macrophages. This may drive M2-like pro-resolving effects in mice with polymicrobial sepsis, suggesting that RvE1 has the therapeutic potential for sepsis through the modulation of macrophages inflammation. It should be noted that the later anti-inflammatory stage of sepsis is the result of a self-regulatory mechanism in response to severe systemic inflammation. Therefore, enhancement pro-resolving effects in macrophage by RvE1 at the early stages of polymicrobial sepsis may prevent later immune suppression.

In line with our *in vivo* observations, we have shown reduced formation of pro-inflammatory cytokines, IL-6 and IL-1β, and chemokine, CCL2, transcripts in LPS-stimulated BMDMs following RvE1 pre-treatment *in vitro*. The secretion of pro-inflammatory cytokines by macrophages is a key driver in the pathophysiology of the cardiac dysfunction in sepsis, as these cytokines decrease the myofibril response to calcium, enhance mitochondrial dysfunction, and downregulate β receptors (1).

Akt is a member of a conserved family of phosphoinositide 3-kinases (PI3K) signal transduction enzymes; activation of which mediates cell survival and growth, and has cardioprotective effects (20, 32). Here we demonstrate that RvE1 treatment preserves Akt-phosphorylation and, hence, activity in the heart of mice with sepsis. Indeed, activation of the pro-survival Akt pathway has been reported to reduce sepsis-induced cardiac dysfunction (15, 33, 34). Our previous studies have shown that activation of Akt results in inhibition of the activation of NF-κB which was associated with attenuated cardiac dysfunction (35) and reduction of acute kidney injury (36) in murine sepsis. Mechanically, the pro-resolving effects of RvE1 are transduced through ChemR23 receptor (37). Indeed, RvE1 regulates Akt phosphorylation through ChemR23, for instance, in human ChemR23-transfected Chinese hamster ovary cells (38). Notably, RvE1 augments Akt activity in the border zone of hearts exposed to ischemia-reperfusion which is associated with a reduced infarct size (26). RvE1 also activates Akt in cardiac cells (H9c2 cardiac embryonic myoblast line) exposed to hypoxia or hypoxia/reoxygenation (26). Thus, we speculate that the activation of Akt induced by RvE1 in the heart contributes to the improvement in cardiac dysfunction caused by sepsis.

NF-κB is one of the most potent pro-inflammatory transcription factors, which consists of heterodimer-subunits p50 and p65 (39). We have demonstrated that RvE1 treatment in mice with polymicrobial sepsis is associated with a robust reduction in IKK α/β phosphorylation and, thus, nuclear translocation of p65 in the heart. These findings are in line with our previous studies which show that activation of NF-κB pathway plays a key role in the pathophysiology of sepsis-induced cardiac dysfunction and that inhibition of NF-κB activation attenuates the cardiac function in sepsis (15). RvE1 has been shown to suppress NF-κB activation in pulmonary macrophages *in vitro* (40). The suppression of NF-κB activation in macrophages may have contributed to our observed reduced pro-inflammatory protein transcripts in RvE1-treated BMDMs. Additionally, NF-κB activation can be induced by the exposure to proinflammatory cytokines, such as IL-1β and TNFα (41). Therefore, reduced pro-inflammatory cytokines leads to decreased NF-κB activation, thus further dampens the inflammatory reaction, which contributes to attenuated polymicrobial sepsis-induced cardiac dysfunction. Moreover, the observed reduction in ERK1/2 and JNK1/2 phosphorylation upon RvE1 treatment may lead to less NF-κB activation and decreased cytokine production, thus generate a positive feedback loop and further reduce the inflammation in the heart (42). Mechanically, it has been shown that RvE1 inhibits TNFα-induced NF-κB activation *via* ChemR23 in HEK293 cells transfected with ChemR23 receptor (37).

SPMs such as RvD1, RvD5, and PD1 enhance phagocytosis and lower antibiotic requirements for bacterial clearance (43). Indeed, in this study, RvE1 treatment leads to improved bacterial clearance in the peritoneal cavity (measured as reduction in bacterial counts). We propose that an increased bacterial clearance by RvE1 will limit inflammation and improve resolution in septic mice. In line with our study, another resolvin, RvD2, has been shown to improve survival rate of septic mice by limiting the number of live aerobic bacteria in both blood and peritoneum (44). Additionally, RvE1 protects mice from bacterial pneumonia and acute lung injury by enhancing clearance of *E. coli* (13). In our study, we have observed an increase in macrophage recruitment in the peritoneal cavity after RvE1 treatment which may have contributed to the improved bacterial clearance. Indeed, our *in vitro* study has shown that addition of RvE1 augmented phagocytosis of *E. coli* in BMDMs. Mechanically, RvE1 enhances phagocytosis in human monocyte-derived macrophages in a ChemR23-dependent manner (38). Similarly, in mice, RvE1 enhances peritoneal macrophage phagocytosis, and these effects are absent in peritoneal macrophages obtained from ChemR23 knockout mice (45). Particularly, ChemR23 is upregulated in human M1 macrophages, and such M1 macrophages show increased phagocytosis in response to RvE1 stimulation, whereas human M2 macrophages do not express the receptor (46).

Interestingly, a recent study has shown that pre-treatment of RvE1 inhibits the infiltration of macrophages into the heart and spleen in mice with LPS-induced cardiac injury (28), whereas our study show that RvE1 increases macrophages number in the peritoneal cavity where live bacteria are released. Zhang et al.'s results compliment the data in the present study; macrophages have an important role in the clearance of bacteria, they recognize bacteria *via* pattern recognition receptors (PRRs), which is essential for macrophage activation, phagocytosis, and bacteria killing by the host (47). Here we have shown RvE1 increases macrophages number in peritoneum associated with an enhanced bacterial clearance, which is of utmost importance in improving the clinical outcome of mice with sepsis. However, it is possible that RvE1 acts differently on macrophages recruitment to loci such as heart and spleen where no bacterium is present, by reducing macrophages infiltration to limit pro-inflammatory responses and preserve organ functions; this merits future investigation. Over-activation of PRRs may cause systemic inflammatory response and elicit harmful damage to the host (47). In our study, although we have seen an increased number of macrophages upon RvE1 treatment, the majority of macrophages are non-pro-inflammatory MHC II^−^ macrophages, indicating that while promoting the phagocytotic activity, RvE1 also reduces pro-inflammatory activation of macrophages. In line with our study, RvD2 treatment also increases peritoneal mononuclear cells in CLP-induced peritonitis, which contributes to a reduced bacterial burden in peritoneal cavity (44).

## Limitations of the Study

We have shown that RvE1 treatment leads to greater cardiac phosphorylation of Akt and reduced activation of cardiac NF-κB, ERK1/2, and JNK1/2 phosphorylation, which is associated with improved cardiac function. However, this *in vivo* study does not allow us to identify specific cell types that are involved in RvE1-induced beneficial changes in cardiac signaling pathways. The pro-resolving effects of RvE1 act through ChemR23 receptor (37) which expresses in macrophages and neutrophils (48). Indeed, our *in vitro* study has shown that RvE1 dampens the inflammatory reaction of BMDMs in response to LPS stimulation. Clearly, cardiomyocytes are the most predominant cell type in the heart, impaired cardiomyocyte contractility directly leads to cardiac dysfunction. Reduced pro-inflammatory cytokines secreted by immune cells upon RvE1 treatment may have improved cardiomyocyte contractility. However, what is more intriguing is that ChemR23 is expressed in murine cardiomyocytes, this reinforces possible direct effects of RvE1 in activation of cardiomyocytic ChemR23 receptor however, further work is needed to confirm this (49).

## Conclusions

This paper shows for the first time that the development of cardiac dysfunction induced by a clinically relevant polymicrobial sepsis model is associated with low levels of SPMs, particularly RvE1 (~93-fold reduction), in the heart. This finding formed the basis for our hypothesis that restoration of RvE1 in polymicrobial sepsis may reduce the cardiac dysfunction. Indeed, therapeutic administration of RvE1 1 h after CLP surgery reduced cardiac dysfunction and improved clinical performance in mice with polymicrobial sepsis receiving analgesics, antibiotics, and fluid resuscitation. RvE1 also aids resolution by increasing MHC II^−^ macrophage recruitment to the peritoneum. More importantly, we have shown that RvE1 treatment enhances phagocytosis of macrophages resulting in a reduced peritoneal bacterial load in mice with sepsis, which is of utmost importance in the therapy of sepsis. We propose that the beneficial effects of RvE1 on septic hearts are secondary to the modulated host immune response, greater cardiac phosphorylation of Akt, and reductions in the activation of cardiac NF-κB, ERK1/2, and JNK1/2. Thus, RvE1 may represent a novel pro-resolving and anti-inflammatory therapeutic approach for the treatment of cardiac dysfunction and assisting bacterial clearance in sepsis.

## Data Availability Statement

The raw data supporting the conclusions of this article will be made available by the authors, without undue reservation.

## Ethics Statement

The animal study was reviewed and approved by the local Animal Use and Care Committee approved animal experiments in accordance with the derivatives of both, the Home Office guidance on the Operation of Animals (Scientific Procedures) Act 1986, and the Guide for the Care and Use of Laboratory Animals of the National Research Council.

## Author Contributions

JC, JD, and CT conceived and designed the experiment. JC, GP, DC, SA, MS, AC, LM, and RC performed the experiments. JC, GP, MC, and JD analyzed the data. JC, CT, MC, and GP contributed to the writing of the manuscript. All authors contributed to the article and approved the submitted version.

## Conflict of Interest

The authors declare that the research was conducted in the absence of any commercial or financial relationships that could be construed as a potential conflict of interest.
