# Correction: RvE1 attenuates polymicrobial sepsis-induced cardiac dysfunction and enhances bacterial clearance

**DOI:** 10.3389/fimmu.2025.1659224

**Published:** 2025-07-31

**Authors:** Jianmin Chen, Gareth S. D. Purvis, Debora Collotta, Sura AI Zoubi, Michelle A. Sugimoto, Antonino Cacace, Lukas Martin, Roman A. Colas, Massimo Collino, Jesmond Dalli, Christoph Thiemermann

**Affiliations:** ^1^ Barts and the London School of Medicine and Dentistry, William Harvey Research Institute, Queen Mary University of London, London, United Kingdom; ^2^ Sir William Dunn School of Pathology, University of Oxford, Oxford, United Kingdom; ^3^ Department of Drug Science and Technology, University of Turin, Turin, Italy; ^4^ Department of Basic Medical Sciences, School of Medicine, Al-Balqa Applied University, As-Salt, Jordan; ^5^ Diabetes Complication Research Centre, School of Medicine, UCD Conway Institute, University College Dublin, Dublin, Ireland; ^6^ Department of Intensive Care and Intermediate Care, RWTH University Hospital Aachen, Aachen, Germany

**Keywords:** polymicrobial sepsis, resolvin E1, bacterial clearance, immune response, cardiomyopathy

There was a mistake in **Figure 3A** as published. The previous image of **Figure 3A** (MHCII vs. SSC-A scatter plot) was selected from a mouse in the 24-hour post-CLP group that had received RvE1 treatment, as we initially understood that RvE1-treated mice were also subjected to 24-hour CLP. However, to avoid any further confusion, we have now replaced this scatter plot with an image from a mouse subjected to CLP without RvE1 treatment. This change more accurately reflects the legend of **Figure 3A**: “(A) Flow cytometry gating strategy of mouse peritoneal immune cells 24 h post-CLP”. The corrected **Figure 3** and its caption appear below.

**Figure 3:**
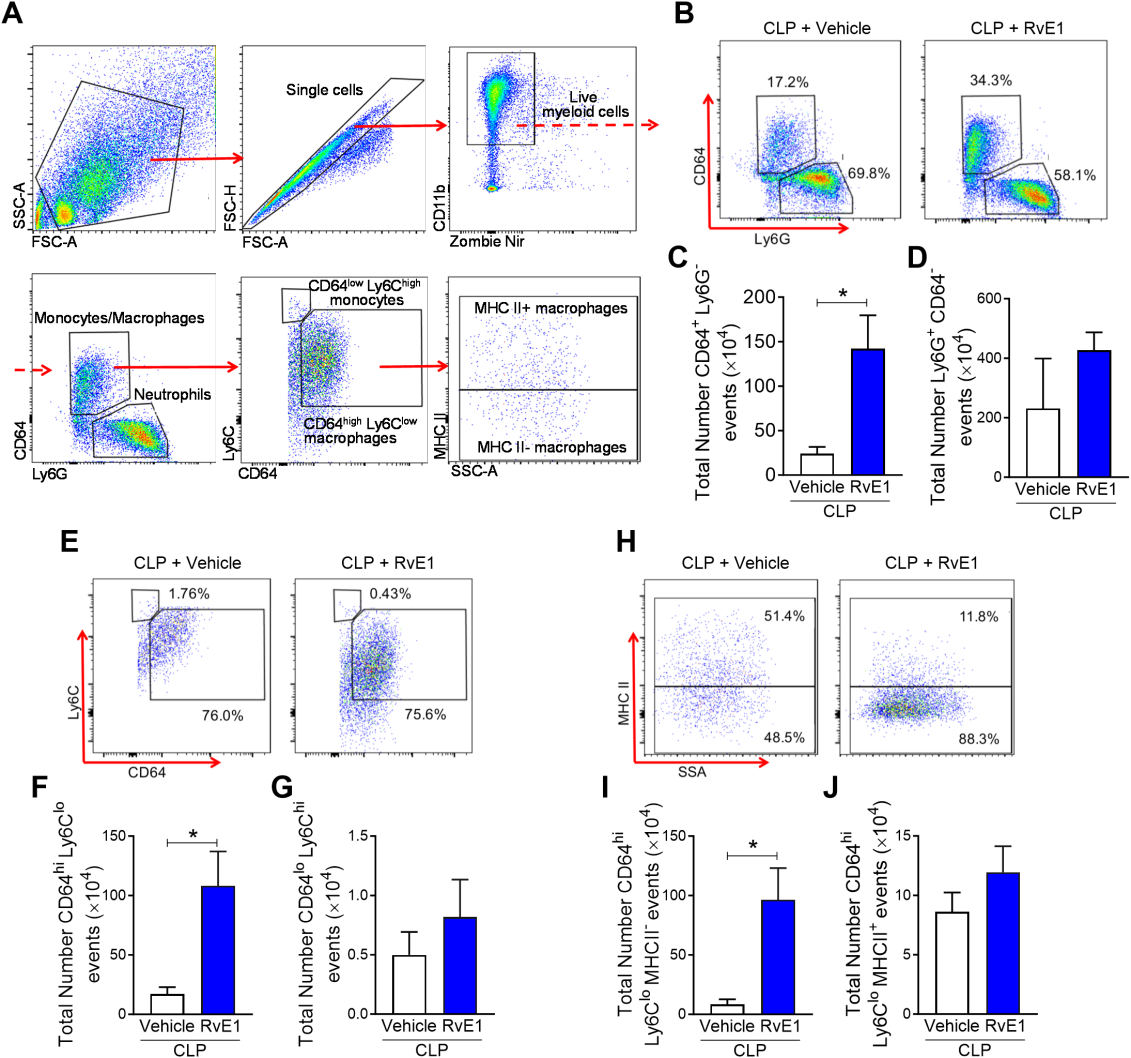
RvE1 treatment enhances MHC II- macrophage recruitment in peritoneal cavity. Mice underwent CLP surgery. One hour after CLP, mice were treated with either RvE1 (1 μg/mouse *i.v.*) or vehicle (100 μl PBS, 0.1% Ethanol). **(A)** Flow cytometry gating strategy of mouse peritoneal immune cells 24 h post-CLP. **(B)** Scattergrams illustrating monocyte/macrophage (identified as Ly6G^-^CD64^+^) and neutrophil (identified as Ly6G^+^CD64^-^) positive events in peritoneal lavages from CLP mice with vehicle or RvE1 treatment. **(C, D)** Cumulative data for peritoneal CD64^+^ monocytes/macrophages and Ly6G^+^ neutrophils. **(E)** Scattergrams illustrating macrophage (identified as CD64^high^Ly6C^low^) and monocyte (identified as CD64^low^Ly6C^high^) positive events in peritoneal lavages from CLP mice with vehicle or RvE1 treatment. **(F, G)** Cumulative data for peritoneal CD64^high^Ly6C^low^ macrophages and CD64^low^Ly6C^high^ monocytes. **(H)** Scattergrams illustrating MHC II^-^ macrophage and MHC II^+^ macrophage positive events in peritoneal lavages from CLP mice with vehicle or RvE1 treatment. **(I, J)** Cumulative data for peritoneal MHC II^-^ macrophages and MHC II^+^ macrophages. Data are expressed as mean ± SEM of four mice for vehicle group and five mice for RvE1 treatment group. Data were analyzed by unpaired Student’s t-test. *P< 0.05 versus CLP + Vehicle group.

