# Supplementary material for: RvE1 Attenuates Polymicrobial Sepsis-Induced Cardiac Dysfunction and Enhances Bacterial Clearance
Source: Front Immunol. 2020 Sep 2;11:2080. doi: 10.3389/fimmu.2020.02080 (PMC7492649; doi:10.3389/fimmu.2020.02080)
Supplement: Supplementary file 1 [file Table_1.DOCX]

Supplementary Material

**RvE1 attenuates experimental polymicrobial sepsis-induced cardiac dysfunction and enhances bacterial clearance**

**Jianmin Chen, Gareth S.D. Purvis, Debora Collotta, Sura Al Zoubi, Michelle A. Sugimoto, Antonino Cacace, Lukas Martin, Roman A. Colas, Massimo Collino, Jesmond Dalli, Christoph Thiemermann**

## Supplementary Table

**Table S1:** Differential regulation of pro-resolving mediators in murine hearts during polymicrobial sepsis induced by CLP.

| **DHA bioactive metabolome** | Lipid mediators levels (pg/heart) | | | | | | | |
| --- | --- | --- | --- | --- | --- | --- | --- | --- |
|  |  |  | Sham | | | CLP | | |
|  | **Q1** | **Q3** | Mean | ± | SEM | Mean | ± | SEM |
| RvD1 | 375 | 215 | 13.7 | ± | 3.3 | 8.0 | ± | 1.8 |
| RvD2 | 375 | 141 | 14.6 | ± | 5.5 | 11.9 | ± | 3.1 |
| RvD3 | 375 | 147 | 9.0 | ± | 2.2 | 5.9 | ± | 1.6 |
| RvD4 | 375 | 101 | 82.4 | ± | 22.5 | 28.9 | ± | 10.1 * |
| RvD5 | 375 | 199 | 7.1 | ± | 2.5 | 4.4 | ± | 1.3 |
| RvD6 | 375 | 159 | 16.3 | ± | 5.0 | 12.8 | ± | 3.9 |
| 17R -RvD1 | 375 | 215 | 47.7 | ± | 11.1 | 9.1 | ± | 2.0 ** |
| 17R -RvD3 | 375 | 147 | 6.7 | ± | 1.2 | 1.3 | ± | 0.3 *** |
|  |  |  |  |  |  |  |  |  |
| PD1 | 359 | 153 | 39.8 | ± | 13.5 | 13.4 | ± | 6.4 |
| PDX | 359 | 153 | 24.7 | ± | 7.1 | 42.9 | ± | 25.9 |
| 17R-PD1 | 359 | 153 | 23.7 | ± | 8.5 | 1.6 | ± | 0.6 * |
|  |  |  |  |  |  |  |  |  |
| MaR1 | 359 | 221 | 246.2 | ± | 69.9 | 38.8 | ± | 9.0 ** |
| MaR2 | 359 | 191 | 12.5 | ± | 3.9 | 9.6 | ± | 2.7 |
| 7S,14S-diHDHA | 359 | 221 | 8.2 | ± | 3.3 | 2.7 | ± | 0.8 |
|  |  |  |  |  |  |  |  |  |
| **n-3 DPA bioactive metabolome** |  |  |  |  |  |  |  |  |
| RvT1 | 377 | 193 | 40.3 | ± | 12.7 | 19.4 | ± | 5.8 |
| RvT2 | 377 | 215 | 32.5 | ± | 7.9 | 3.0 | ± | 0.8 ** |
| RvT3 | 377 | 143 | 7.9 | ± | 1.6 | 1.3 | ± | 0.3 *** |
| RvT4 | 361 | 193 | 17.4 | ± | 4.4 | 18.4 | ± | 6.4 |
|  |  |  |  |  |  |  |  |  |
| RvD1_n-3 DPA_ | 377 | 143 | 32.4 | ± | 8.5 | 5.2 | ± | 1.2 ** |
| RvD2_n-3 DPA_ | 377 | 233 | 7.6 | ± | 1.5 | 2.7 | ± | 0.4 ** |
| RvD5_n-3DPA_ | 361 | 199 | 5.3 | ± | 1.3 | 4.6 | ± | 1.4 |
|  |  |  |  |  |  |  |  |  |
| PD1_n-3 DPA_ | 361 | 183 | 54.1 | ± | 14.6 | 31.6 | ± | 8.3 |
|  |  |  |  |  |  |  |  |  |
| MaR1_n-3 DPA_ | 361 | 223 | 12.9 | ± | 6.5 | 2.5 | ± | 0.6 |
|  |  |  |  |  |  |  |  |  |
| **EPA bioactive metabolome** |  |  |  |  |  |  |  |  |
| RvE1 | 349 | 195 | 251.9 | ± | 89.5 | 2.7 | ± | 1.0 ** |
| RvE2 | 333 | 199 | 3.9 | ± | 1.7 | 2.1 | ± | 0.5 |
| RvE3 | 333 | 201 | 25.4 | ± | 10.1 | 2.1 | ± | 0.5 * |
|  |  |  |  |  |  |  |  |  |
| **AA bioactive metabolome** |  |  |  |  |  |  |  |  |
| LXA_4_ | 351 | 115 | 54.8 | ± | 11.2 | 27.0 | ± | 8.6 |
| LXB_4_ | 351 | 115 | 160.8 | ± | 26.3 | 51.5 | ± | 17.4 ** |
| 15R-LXA_4_ | 351 | 115 | 9.5 | ± | 1.6 | 27.0 | ± | 8.6 |
| 15R-LXB_4_ | 351 | 221 | 14.7 | ± | 5.0 | 4.6 | ± | 0.9 * |
| 5S,15S-diHETE | 335 | 235 | 1473.7 | ± | 473.0 | 239.9 | ± | 50.7 |
|  |  |  |  |  |  |  |  |  |
| LTB_4_ | 335 | 195 | 88.7 | ± | 26.7 | 31.0 | ± | 6.7 * |
|  |  |  |  |  |  |  |  |  |
| PGD_2_ | 351 | 189 | 72.5 | ± | 22.3 | 71.9 | ± | 19.3 |
| PGE_2_ | 351 | 189 | 120.7 | ± | 15.1 | 176.8 | ± | 76.4 |
| PGF_2a_ | 353 | 193 | 98.3 | ± | 9.3 | 100.7 | ± | 12.0 |
| TXB_2_ | 369 | 169 | 87.9 | ± | 23.2 | 72.2 | ± | 24.4 |

Mice were subjected to CLP or sham-operated surgery and heart tissues were collected after 24 h. Tissues were then homogenised and mediators were identified and quantified using LC-MS/MS based lipid mediator profiling. Results are expressed as pg/heart. n= 5 for sham group and 7 for CLP group. Data are mean ± SEM and were analysed by unpaired Student’s t-test. **P*<0.05, ***P*<0.01, ****P*<0.001 vs. Sham group.
